# Balancing misclassification errors in image-based inference using problem domain semantics and a nested cascade architecture

**DOI:** 10.1007/s00521-025-11613-8

**Published:** 2025-09-13

**Authors:** Xin Du, Rajesh Jena, Katayoun Farrahi, Mahesan Niranjan

**Affiliations:** 1 RadNet Data Science Team, The Cavendish Laboratory, University of Cambridge, Cambridge, UK; 2 Vision, Learning and Control Group, Electronics and Computer Science (ECS), University of Southampton, Southampton, UK; 3 The Department of Oncology, University of Cambridge, Cambridge, United Kingdom

**Keywords:** Nested cascade architecture, Multi-class classification, Domain semantics, Misclassification errors

## Abstract

Pattern recognition models, particularly neural networks, often focus on maximising classification accuracy. However, in practice, the types of errors made (misclassification between different classes) can have varying associated costs. Current methods overlook varying misclassification error types. Misclassification labels can either be available from expert knowledge or derived from semantics of textual descriptions of class labels. Exploiting such misclassification costs can have significant implications when deploying machine learning systems. Here, using five examples from image and tabular domains, we show how a deep neural architecture trained in a nested layer-wise fashion (cascade learning) in which early layers solve easier problems than later ones could exploit such hierarchical aspects of class labels. We employ a measure of performance called “severity” of errors and show how emphasis could be placed on classes that are deeper in the hierarchy, ignoring errors that arise between semantic neighbours.

**Supplementary Information:**

The online version contains supplementary material available at 10.1007/s00521-025-11613-8.

## Introduction

Nowadays, pattern classification problems, solved to very high performance using recent developments in machine learning, permeate many aspects of life in science and society. A particular example is the inference of multi-class targets from images, including the diagnosis of medical conditions from radiographs. Training such systems is usually done by minimising a loss function which serves as a proxy for classification accuracy. When maximising classification accuracy (or equivalently minimising error rates), one would set a precise operating point when the costs of associated misclassifications are known a priori [1]. When this is not the case, it is usual to maximise the area under a receiver operating characteristics (ROC) curve, integrating over all possible false positive rates.

This paper is based on the premise that in many practical multi-class classification problems, additional exploitable information is available either in the structure or linguistic description of the classes themselves. Let us consider the problem where the goal is to detect one of several diseases from chest radiographs (an example considered for empirical evaluations later in this paper). Two of these diseases may be very similar in terms of the clinical treatment plan and outlook for the patient. If so, the error of a classifier that confuses these two is of low clinical importance. In contrast, consider another disease, a very different clinical outlook and mode of treatment. Confusing this disease with either of the former two cases would have severe consequences for the patient. Similarly, if we imagine a self-driving car making decisions based on vision, confusing a lamp post as a tree is of little significance in comparison with confusing it as a person crossing the road. What follows is an inherent hierarchical structure in class labels with potentially different costs associated with particular misclassification.

The hierarchical structure may be derived from prior knowledge of what one does with the classification decisions or, as we show later in this paper, derived by analysis of the natural language terms used to describe the classes. Once we have such a hierarchy, loss functions minimised in the training process should be weighted by the corresponding costs incurred (if known). One finds such is rarely the case in mainstream machine learning literature although several of the problems considered in the community indeed have such hierarchies.

In this paper, we offer an architectural and algorithmic strategy, referred to as Nested Semantic Cascade Learning (NSCL) to address this issue of detecting and exploiting hierarchies in multi-class labels. We build on an established layer-wise training approach (Cascade Learning (CL) [2]) organising the multi-class classification problem to be solved in a coarse to fine manner. Before training the models, a hierarchical dendrogram is constructed based on the semantic relationships among labels, leveraging pre-trained word-to-vector language models. This dendrogram serves as a structured representation of the semantic context, enabling the organisation of labels into a hierarchy of coarse and fine clusters. These clusters are then strategically allocated across different stages of the model to optimise learning dynamics.

The process of constructing a dendrogram provides several advantages. First, it captures the inherent relationships among labels by embedding them into a continuous vector space where semantic similarity is preserved [3, 4]. This allows the model to exploit shared features across related labels, particularly for classes with limited data. Second, the hierarchical organisation aids in progressive learning, where the model learns coarse-level distinctions at early stages before refining its understanding at finer levels. This approach mirrors human cognitive processes, where general categories are identified before distinguishing specific subcategories [5].

Thus, early layers in the framework solve simpler classification problems with later ones discriminating between harder fine-grained classes. The contributions we make in this paper are as follows:

- We work with a performance measure called severity, that scores the performance of the multi-class classifier and is sensitive to the hierarchy of classes.
- Our method incorporates cluster hierarchy by including multi-class information in a coarse to fine manner derived from a distributed vector representation. Where such hierarchies are available from prior knowledge, they are incorporated into our approach.
- We build on CL [2] to impose a nested class structure deep learning pattern classifier in which early layers solve simpler (few classes) and later ones solve harder problems (several classes).
- We evaluate our proposed methodology on three vision tasks (CIFAR10, CIFAR100 and CheXpert) and two tabular tasks (Obesity and Bacteria), with NSCL showing consistently lower severity of mistakes on all datasets considered.

## Related work

**Cascade learning.** Drawing inspiration from Fahlman and Lebiere [6], Marquez et al. [2] introduced Deep CL a strategy to train deep neural networks layer by layer, primarily motivated by computational savings over end-to-end (E2E) training. The work due to Belilovsky et al. [7] showed that such layer-wise training can scale to very large problems such as ImageNet [8], achieving state-of-the-art performance. A study based on how information compression in learning [9] applies to CL was done in [10]. Further Wang et al. [11] showed that apart from computational savings and performance, layer-wise training induces a coarse-to-fine feature extraction ability in networks. This property has been demonstrated to effectively extract localised features, making it particularly valuable for explaining medical decisions based on radiographic images. The usefulness of such hierarchically extracted coarse-to-fine features in transfer learning was demonstrated by Du et al. [12] using a human activity recognition problem, where coarse features not too specific to a source domain were of better use in transfer to a target domain.

**Hierarchy in images.** Datasets with hierarchical labels, such as CIFAR10 and CIFAR100, have been widely utilised in computer vision research to explore multi-class classification frameworks. These datasets, while smaller in scale compared to medical datasets, offer opportunities to study how hierarchical relationships among labels can improve model performance. Similarly, the CheXpert dataset [13], consisting of over 200, 000 images from 60, 000 patients, with annotations of 14 disease types and being publicly available has attracted much attention. In addition to class labels, diseases in the dataset are specified in the form of a directed acyclic graph (DAG). A five-class problem using the more prevalent diseases, cast as five binary classification problems, is often the problem considered by authors [14, 15], including a critical evaluation of transfer learning to medical contexts [16]. Despite the richness of the DAG being made available [17], surprisingly, very few attempts have been made in the literature to take advantage of label hierarchies, in this dataset or elsewhere in the literature. Pham et al. [18] and Srivastava and Mishra [19] are exceptions wherein the authors introduce a hierarchical loss to an E2E learning framework, whereas Pham et al. [18] were proposing a two-stage training procedure.

**Hierarchical classifier.** In the context of general image classification, Bertinetto et al. [20] introduce a hierarchical cross-entropy loss function to work with the ImageNet visual scene classification, something very few other authors appear to have attempted, despite ImageNet having a clear label hierarchy. Karthik et al. [21] further emphasise mitigating severe classification errors using adversarial training mechanisms informed by semantic knowledge. By incorporating hierarchical loss functions and semantic similarity measures into adversarial frameworks, the authors aim to align predictions with structured label spaces, ensuring that even when misclassifications occur, they are semantically meaningful (i.e. between related classes).

Helus et al. [22] also introduce a method to reduce mistake severity in deep learning models by manipulating prediction likelihoods during inference. The approach utilises hierarchical semantic relationships, applying post hoc adjustments to predicted probabilities to align them with a structured label space. Without requiring additional training, the method minimises severe mistakes by prioritising semantically similar classes when errors occur. In the domain of self-supervised learning (SSL), a particularly elegant piece of work due to Ben-Shaul et al. [23] relates features extracted by SSL to align to semantic classes.

Building on these works, Srivastava and Mishra [19] introduced an evaluation framework for several state-of-the-art models using a severity metric based on the Lowest Common Ancestor (LCA) distance [21]. This metric quantifies the severity of classification errors by measuring the semantic distance between misclassified and true labels within a hierarchical taxonomy. The models evaluated in this framework were trained by optimising hierarchical loss functions. Their approach demonstrated improved error interpretability by aligning model predictions with the hierarchical structure, providing a more nuanced understanding of model performance compared to traditional flat metrics.

Furthermore, Li et al. [24] extended the application of hierarchical loss functions to semantic segmentation of images, demonstrating their utility in tasks that require the preservation of semantic consistency in spatially complex outputs. These contributions from a small number of examples attempt to exploit class hierarchies.

In the above space of present research, we take a very different approach by casting the problem of exploiting semantic hierarchy in classes on a cascade-trained neural network architecture. The innovative step in our work, as distinct from previous work, is to construct a hierarchy of problems to solve inspired either by available domain knowledge or by semantic clustering of the class labels, in a continuous representation space. This gives us a coarse-to-fine learning setting in which, as noted earlier, early layers of a network solve simpler problems with a few classes followed by later layers solving finer-grained problems.

**Fig. 1:**
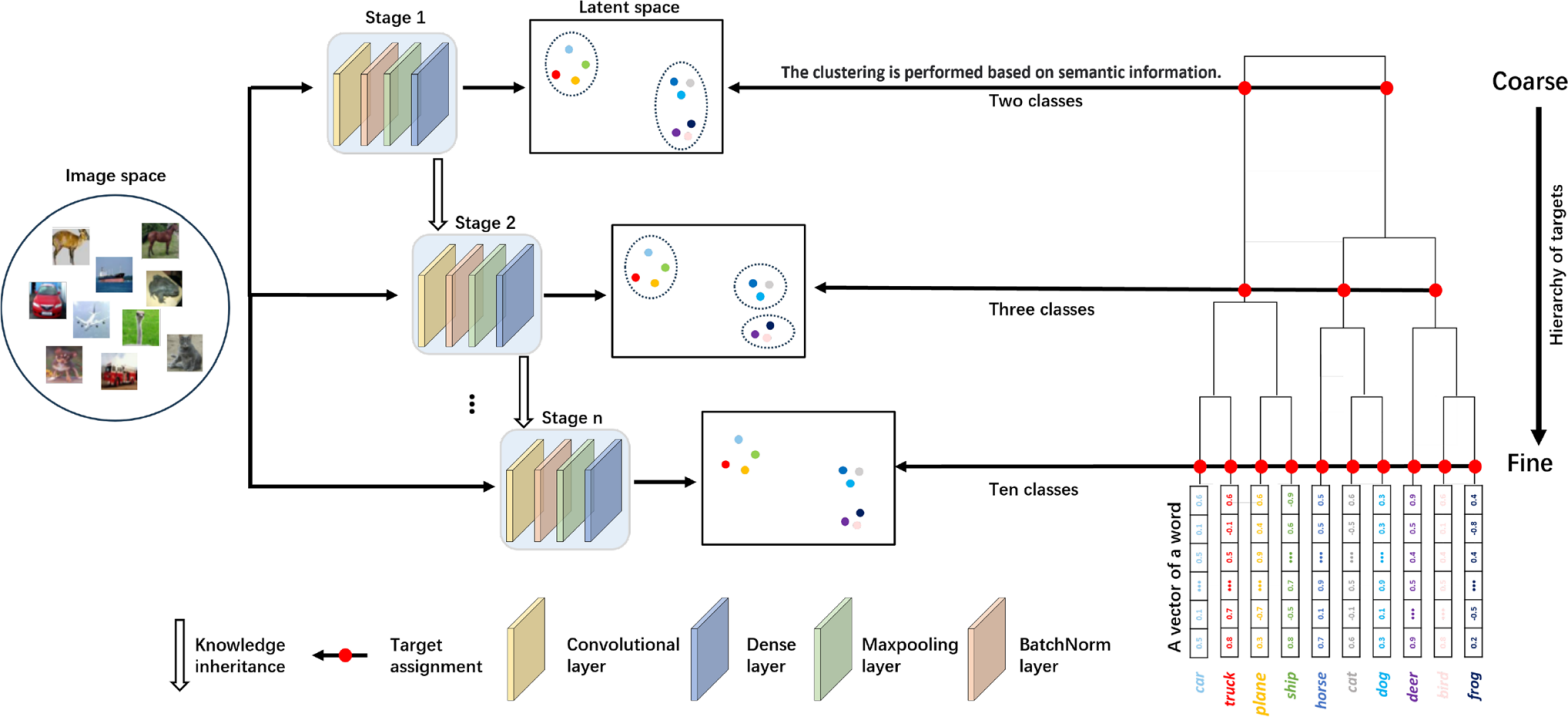
Schematic diagram of semantic cascade learning (SCL). At each level of the hierarchy, a neural network is trained to solve a multi-class classification task, each layer classifying progressively a larger number of classes. The number of classes each layer targets is derived either from prior knowledge of the problem domain (e.g. the directed acyclic graph of diseases in the CheXpert dataset), or by hierarchical clustering of continuous representations of the text describing class labels, thus deriving a coarse to fine classification system. The figure shows an example from CIFAR10, clustered into a three-level hierarchy, with the red dots representing the classes at each stage. The latent space visualisation illustrates the distribution of semantic embeddings following dimensionality reduction

## Approach

### Learning architectures

Motivated by the approaches in [2, 11, 12], we investigate CL—including SCL and nested learning—to capture class hierarchies, and we train E2E models, including hierarchical E2E learning, to provide benchmarking baselines.

#### Semantic cascade learning (SCL)

Figure 1 shows the framework of exploiting class hierarchies in a CL setting. A class hierarchy is either available from prior knowledge or derived by cluster analysis (such as distributed embeddings obtained from Word2Vec [3] or Biowordvec [4]). Cutting the dendrogram at different places, simpler and more difficult problems can be extracted. The figure shows three levels of the hierarchy with the number of classes set to two, three and ten derived from the CIFAR10 dataset. We train early parts of the neural network on the simpler problem with a few classes and deeper parts with increasing numbers of classes. This approach differs from classical cascade training, in which the network is typically decomposed into a feature extractor followed by an auxiliary classifier that repeatedly attempts to solve the same classification task at each stage. When going from one layer to the next, the feature extractor is frozen and the newly added layer and auxiliary classifier are initialised and trained using gradient descent. This will form the SCL setting.

In more detail, semantic information of labels can be represented as a hierarchical structure in the form of a tree, denoted by $\zeta =(\Upsilon ,\Lambda )$. Each node $\upsilon \in \Upsilon$ represents a semantic cluster/class/concept, while each edge $(\kappa ,\upsilon ) \in \Lambda$ defines the decomposition relationship between two classes $\kappa ,\upsilon \in \Upsilon$. Specifically, it signifies that a parent node $\upsilon$ is a more general superclass of its child node $\kappa$, for example, $(\kappa ,\upsilon )=(\text {car},\text {vehicle})$. The relationship between $\kappa$ and $\upsilon$, both belonging to set $\Upsilon$, is determined based on the distance between their word embeddings in semantic space. The root node of hierarchy $\zeta$, denoted as $\upsilon ^r$, represents the most general concept. On the other hand, leaf nodes from level *l*, denoted as $\Upsilon ^l=\{\text {car},\text {truck},...\}$ in this case, represent highly specific classes.

For a SCL network, a feature extractor $f_{FE}^l(\mathbf{{I}})$ at level *l* is firstly adopted to map *m* input images $\textbf{I}$ into a dense feature matrix $\mathbf{{R}}=f_{FE}^l(\mathbf{{I}}) \in {\mathbb {R}}^{m\times u}$, where $\textbf{r} \in R$ is the latent representation of image *I*, and *u* denotes the dimension of word embeddings. A classifier $f_C^l$ is then employed to obtain a score map $\mathbf{{Y}}^l=softmax(f_C^l(\mathbf{{R}})) \in [0,1]^{m\times |\Upsilon ^l |}$. Given a score vector $\mathbf{{y}}^l=[y_{\upsilon ^l}]_{\upsilon ^l \in {\Upsilon ^l}}\in [0,1]^{|\Upsilon ^l |}$ and the ground-truth leaf target $\hat{\upsilon }^l \in \Upsilon ^l$, the categorical cross-entropy loss is optimised by minimising ${\mathcal {L}}_{CCE}(\mathbf{{y}}^l)=-\log (y_{\hat{\upsilon }^l})$. To address class imbalance in each dataset, the cross-entropy loss, ${\mathcal {L}}_{CCE}$, is weighted according to the proportion of points from each class. To further incorporate hierarchical semantic information, a regression loss is also applied by minimising distances between latent representations $\mathbf{{R}}$ and word embeddings $\mathbf{{W}} \in {\mathbb {R}}^{m \times u}$. The regression loss in this study is based on the cosine distance $D_c$ between each word embedding vector $\textbf{w} \in W$ and the latent representation vector $\mathbf{{r}}$ of each image, as demonstrated in Eq. (1), following inspiration from Barz and Denzler [25].

1 $$\begin{aligned} {\mathcal {L}}_{R}(\mathbf{{w},r})=\frac{1}{m}\sum _{i=1}^{m}{(1-D_c(\mathbf{{w}}_{i},\mathbf{{r}}_{i}))} \end{aligned}$$

At each level *l*, the regression loss compels models to incorporate semantic information derived from language models to extract latent representations, thereby integrating contextual cues from region-specific literature into image classification instead of relying solely on image characteristics. Consequently, both loss functions are weighted by respective hyperparameters and then combined into an objective function definition, as illustrated in Sect. 3.1.4. For each layer/level, the same objective function is optimised, while inputs of level $l+1$ inherit knowledge from level *l* and the number of nodes $|\Upsilon ^{(l+1)} |$ is greater than $|\Upsilon ^l |$ at level *l*. A further illustration of differences among SCL, CL and E2E learning mechanisms is shown in Figure A1 in section A.

#### Nested learning

Building on the cascade learner, in nested learning we do not freeze previously learned layers and allow them to be fine-tuned by gradient descent as well. For empirical evaluation, we consider nesting with and without the use of semantic information, namely NSCL and Nested CL (NCL). The difference between NCL and E2E learning is NCL utilises pre-trained layers while E2E does not. For completeness, we also consider grouping the classes into random groups of the same membership sizes as semantically clustered groups, referred to as Random Hierarchical Cascade Learning (RHCL).

#### Hierarchical end to end learning (HE2E)

To integrate label information and classifier training in an E2E setting, we use a specific loss function forming a weighted combination of classification loss and a regression loss derived from the vector representation of the class labels. This is defined in the next subsection (Eq. (2)).

#### Loss function

The objective function in our work, as previously mentioned in Sect. 3.1.1, consists of two parts: regression and classification (refer to Eq. (2)). Both parts are weighted by hyperparameters $\alpha$ and $\beta$, allowing for the optimal balance through the combination of learning mechanisms. In our experiment, the parameter $\alpha$ is systematically varied from 0 to 1, with an increment of 0.2, while the parameter $\beta$ is explored within the range of 0 to 7, with a step size of 1. Specifically, when $\alpha <1$, we set $\beta =1-\alpha$; otherwise, we fix $\beta =1$ and allow $\alpha$ to vary in range 1 to 7.

2 $$\begin{aligned} {\mathcal {L}}=\alpha \times {\mathcal {L}}_{R}(\mathbf{{w},r})+\beta \times {\mathcal {L}}_{CCE}(\mathbf{{y}}^l) \end{aligned}$$

### Evaluation metrics

To quantify and compare the difference in performance of the methods, we use three metrics: accuracy, saliency maps [26], and proposed severity, with the specific definition of severity provided in Sect. 3.2.1.

#### Severity

The quality of predictions is evaluated by calculating the severity layer by layer, which is determined by the number of hierarchical levels (*L*) provided by label built-in semantic information. Starting from the root and going down to the leaf nodes, each data point (*I*) at each hierarchical level (*l*) compares its current level prediction ($\upsilon ^l$) with the corresponding target value ($\hat{\upsilon }^l$). If they do not match, the severity of the current level ($S_l$) is assigned a value of 1, otherwise it is assigned a value of 0, as shown in Eq. (3). To normalise across all levels, the severity for each data point is divided by the total number of levels resulting in a definition of severity as shown in Eq. (4). Accordingly, the severity of a classifier (*f*) is represented as the expectation of all severities over data points (*P*(*X*, *Y*)) passed into the classifier as inputs. This is denoted by Eq. (5). According to the definition mentioned above, severity can be employed as a metric to quantify the overall number of misclassified cases across various levels ranging from broader categories to more specific ones, thereby facilitating the evaluation of mistake severity. A higher severity indicates that errors occur predominantly at earlier stages (broader categories) and carry greater significance.

3 $$\begin{aligned} S_l = {\left\{ \begin{array}{ll} 0\text {, } & \upsilon ^l=\hat{\upsilon }^l\\ 1\text {, } & \upsilon ^l\ne \hat{\upsilon }^l \end{array}\right. } \end{aligned}$$

4 $$\begin{aligned} \textrm{severity}=\frac{1}{L}\sum _{l=1}^{L} S_{il}(\upsilon ^l,\hat{\upsilon }^l) \end{aligned}$$

5 $$\begin{aligned} X_s^{(f)} = {\mathbb {E}}_{x_i, y_i \sim P(X, Y)} \left[ \textrm{severity}(y_i,f(x_i)) \right] \end{aligned}$$

Our method offers a task-specific approach to defining hierarchical levels, as opposed to relying on a fixed tree structure used in traditional LCA-based methods [19, 20]. While LCA approaches calculate error severity by determining the distance to the common ancestor for each pair of classes, our method assesses severity by traversing from the root to leaf nodes in a hierarchy defined according to the task requirements. Importantly, it halts further computation if an error occurs at any intermediate level, as errors at higher levels inherently propagate downward. This targeted approach reduces computational overhead, offering greater efficiency and adaptability for complex tasks.

#### IOU of saliency maps

To evaluate the effectiveness of saliency maps, we use the intersection over union (IOU) [11, 27] between masks derived from saliency maps and radiologist annotations. Masks are generated by applying a threshold to convert the darkest regions of saliency maps into binary masks, following the approach proposed by Saporta et al. [15]. Additionally, we assess both coverage accuracy (intersection over annotations) and coverage precision (intersection over masks) to evaluate the location accuracy and area precision provided by saliency maps.

### Datasets and architectures

A total of five datasets were utilised for the empirical analysis, encompassing both image and tabular datasets. Among the image datasets, CIFAR-10 and CIFAR-100 [28] are widely used computer vision benchmarks containing natural images, while CheXpert is a medical imaging dataset specifically designed for chest X-ray analysis.

The tabular datasets include Obesity [29] and Bacteria [30]. The Obesity dataset contains records for estimating obesity levels in individuals from Mexico, Peru, and Colombia, based on their eating habits and physical condition. It consists of 17 attributes and 2,111 records. The Bacteria dataset aims to classify 10 different bacterial species using genomic sequencing data, represented by 287 attributes.

For the image datasets, preprocessing steps include resizing inputs to 224$\times$224 pixels using random cropping, followed by random horizontal flipping and normalisation with mean values (0.485,0.456,0.406) and standard deviations (0.229,0.224,0.225). For the tabular datasets, the raw downloaded data were normalised to range from 0 to 1 for model training without applying any augmentation techniques.

Our convolutional neural network architectures are designed to be relatively simple, with few layers and minimal fine-tuning optimisations without the aim of achieving state-of-the-art results. Notably, for the same medical problem, Raghu et al. [16] conducted experiments using a large ResNet backbone and transfer learning but reported marginal or no performance improvement. In contrast, Wang et al. [11] achieved comparable performance to the aforementioned approach by employing much simpler E2E-trained architectures in both single-stage and cascade settings.

For the CIFAR problems, a simple model consisting of convolutional layers is employed to perform a ten-class classification task using different learning mechanisms. In the E2E, CL, and RHCL mechanisms, the model gradually extends by adding one convolutional layer with 256 units at each level until reaching a final nine-layer model. The difference between CL and E2E lies in the trainable setting of pre-trained layers. For the SCL mechanism, a convolution layer of the same size is used at each stage to construct a four-layer model with class numbers following the sequence two, three, eight, and ten. Based on the settings for CL and SCL mechanisms, corresponding nested learning (NCL and NSCL) is further constructed. All models utilise Adam optimiser [31] with an initial learning rate search ranging from 0.1 to 0.001; after each convolution layer, a max-pooling layer with kernel size 2, stride 2, and zero padding is added; batch normalisation layers [32] follow each max-pooling layer along with ReLU activation function applied to all models. The final output block consists of a fully connected layer with 300 units (equal to word embedding dimension), followed by ReLU activation function and another fully connected layer with a softmax activation function. Each layer sets an initial training period of 50 epochs. The same network structure and training settings are applied to training on CIFAR100 except that SCL and NSCL employ a nine-layer model where class numbers for each level follow this sequence: 2, 4, 8, 16, 32, 64, 80, 90, and 100.

For the CheXpert problem, we focus on six clinically significant and prevalent pathologies [18], namely Health, Cardiomegaly, Edema, Pleural Effusion, Atelectasis, and Consolidation as identified by Raghu et al. [16]. The pathology labels are automatically derived by analysing radiography reports associated with the images; however, a significant number of labels are marked as uncertain. To mitigate the impact of uncertain cases During model training, instances with uncertainty labels are filtered out along with those exhibiting multiple pathologies. Consequently, we construct a dataset consisting only of instances labelled with a single certain disease. This study utilises a total of 78, 034 chest X-rays selected from the original train set and 91 chest X-rays from the original public validation set as the train and test sets, respectively. The backbone architecture consists of a four-layer CNN with layer sizes in sequence: 512, 256, 256, and 128. Additional layers will be added to accommodate different learning mechanisms according to their respective rules. The output block comprises a dense layer with 200 units (determined by medical word embedding dimension [4]), followed by a ReLU activation function and an output dense layer with six units. Each convolutional layer is accompanied by max-pooling layers, batch normalisation layers, and ReLU layers; furthermore, average pooling using a kernel size of $5\times 5$ is applied between the convolutional layer and output block to reduce model parameters caused by full connection. In both SCL and NSCL, the number of classes in each stage follows a sequence: two, four, six, and six. All models are optimised using Adam with an initial learning rate of 0.0001, and training epochs for each layer are set as 35, 40, 45, and 50. The inputs of all models are augmented with random cropping and horizontal flipping to achieve a size of $224*224$.

For the Obesity and Bacteria datasets, 1D convolutional neural networks (CNNs) with four layers are employed. The layer widths are configured sequentially as 16, 32, 64, and 32. To mitigate overfitting, a dropout layer is incorporated into the architecture. Model performance is evaluated using fivefold cross-validation, ensuring robust and unbiased validation. The training process is optimised with a learning rate of $1e-4$ and a batch size of 128, striking a balance between computational efficiency and convergence stability.

**Fig. 2:**
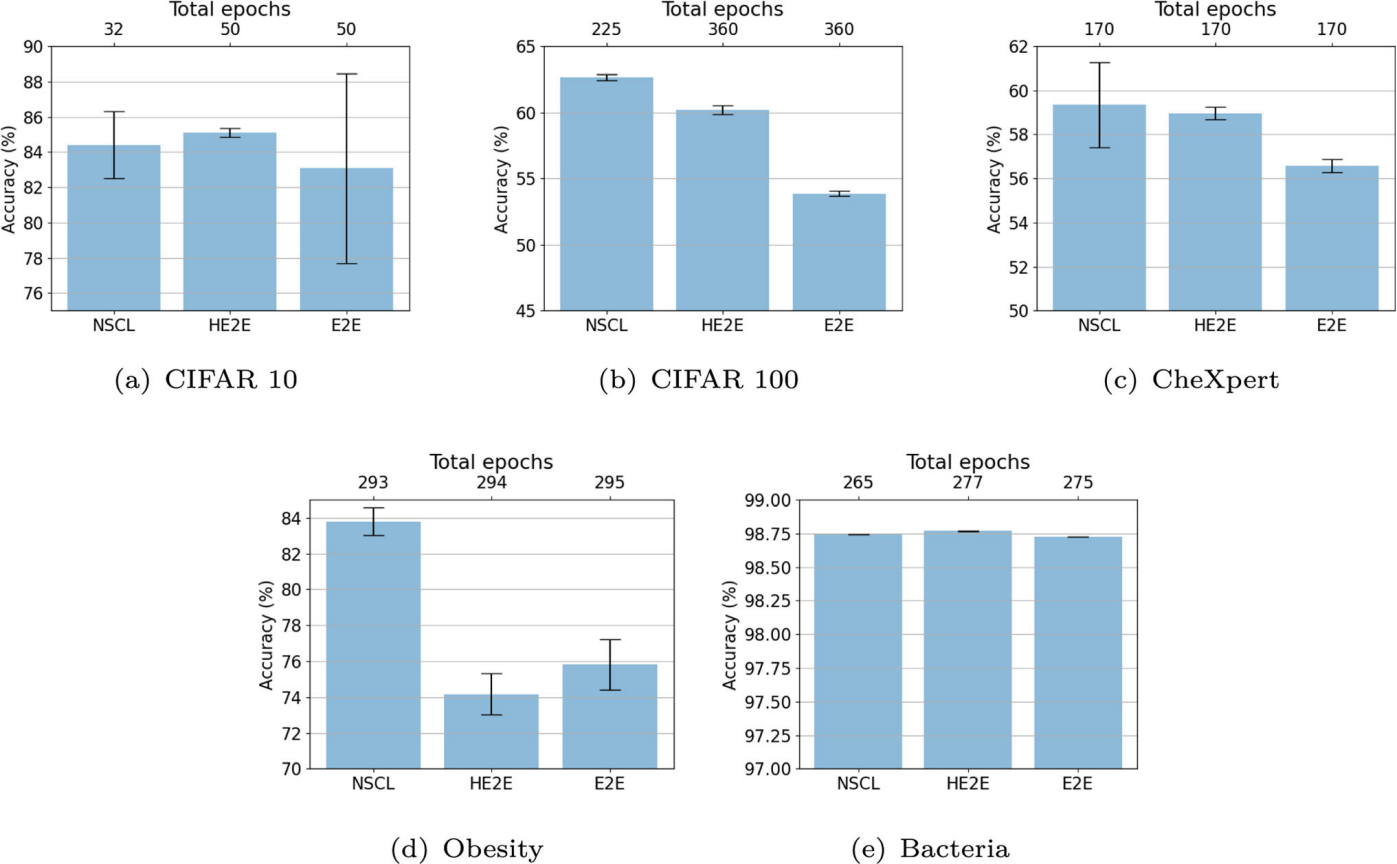
Comparison of classification accuracies for three different methods of training: Vanilla E2E, E2E with a hierarchical loss (HE2E) and the Nested Semantic Cascade Learning (NSCL) proposed in this work. Note our objective is not to outperform state-of-the art in these benchmark problems which have been achieved by extensive hyperparameter tuning. Our results are still comparable to published results with simpler techniques: $78.9\%$ on CIFAR 10 [33] and $52.7\%$ on CIFAR100 [34]. Demonstrating NSCL on architectures and problems with decent baseline performance is the goal in this paper. The numbers of training epochs needed to reach the quoted performance are also displayed above the figures

**Fig. 3:**
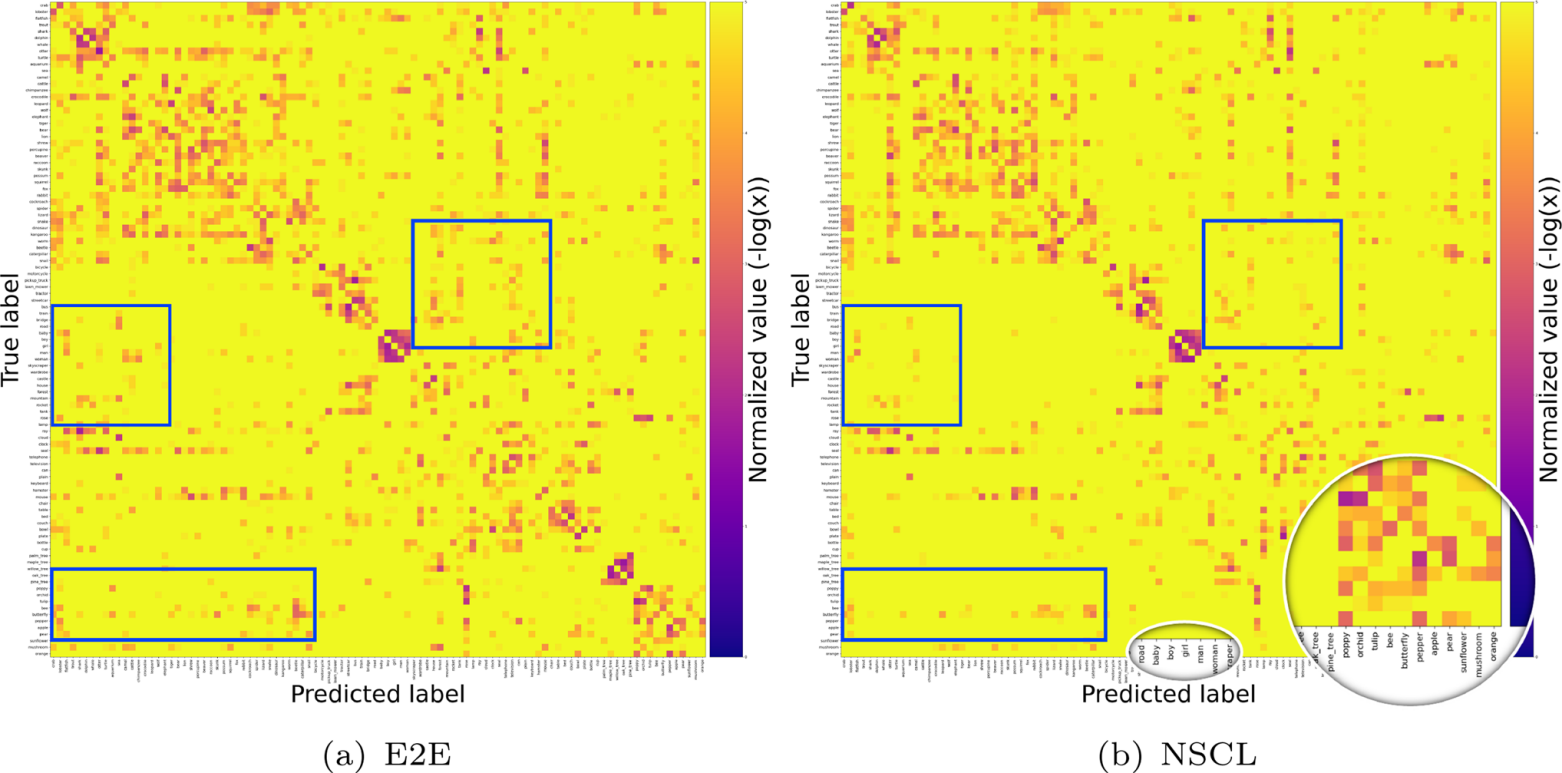
Comparing confusion matrices of E2E and NSCL models on the CIFAR100. For clarity in illustrating the distribution of errors, we have set the diagonals (correct classifications to zero). NSCL generates a more block structured error distribution, with its confusions staying within semantically similar groups of labels. Magnifiers focus on labels with small semantic distance as an illustrative example. A zoom-out version is shown in section A Figure A7

### Accuracy and confusion matrices

Figure 2 shows classification accuracies of three methods (E2E, HE2E and NSCL) on five different multi-class classification problems (CIFAR10, CIAR100, CheXpert, Obesity and Bacteria). Note vanilla E2E performance on the CIFAR problems are not expected to achieve the state-of-the-art performance reported in the literature because they include several steps of careful optimisation and tuning of hyperparameters. For our purpose of illustrating the effect of label hierarchies and semantic information, however, these results are in an acceptable league compared with results published in $78.9\%$ for CIFAR 10 [33] and $52.7\%$ for CIFAR100 [34], which do not include extensive fine-tuning. The bacterial gene expression data are a relatively easy task; hence, all three methods perform well (see Fig. 2e). Note that setting up the problem in a hierarchical fashion has not worsened the performance of this easy problem. Thus, we have a setting which is satisfactory for the purpose. More comparisons with other mechanisms can be found in Figure A2 in section A.1.

Figure 3 shows two confusion matrices for E2E and NSCL on the 100-class CIFAR100 problem. The classes here are ordered to be grouped by the class hierarchy inherent in this dataset. For clarity in the image, we have set the diagonal entries (correct classification) to zero, normalise the error counts and plot its negative log as intensity of the images. What we observe is an approximate block diagonal structure indicating that errors made by the classifiers are in general high within semantically similar groups. With NSCL, we observe this block diagonal structure in error distribution is enhanced and more of the errors are being confined to semantically similar subgroups. The comparison of confusion matrices generated by the E2E and NSCL models reveals distinct error patterns between the two approaches. Highlighted regions located far from the diagonal line indicate instances where classes with minimal semantic connections are being misclassified, representing errors of higher severity. These severe misclassifications are more prevalent in the E2E model compared to the NSCL model. In contrast, misclassifications closer to the diagonal line typically involve classes with stronger semantic connections, suggesting less critical errors. This analysis underscores the differences in the models’ ability to preserve semantic coherence during classification. A further visualisation of the distribution of correctly classified cases is presented in Figure A8, highlighting that NSCL significantly aids in identifying clear boundaries between coarser clusters, whereas E2E struggles to achieve this.

**Fig. 4:**
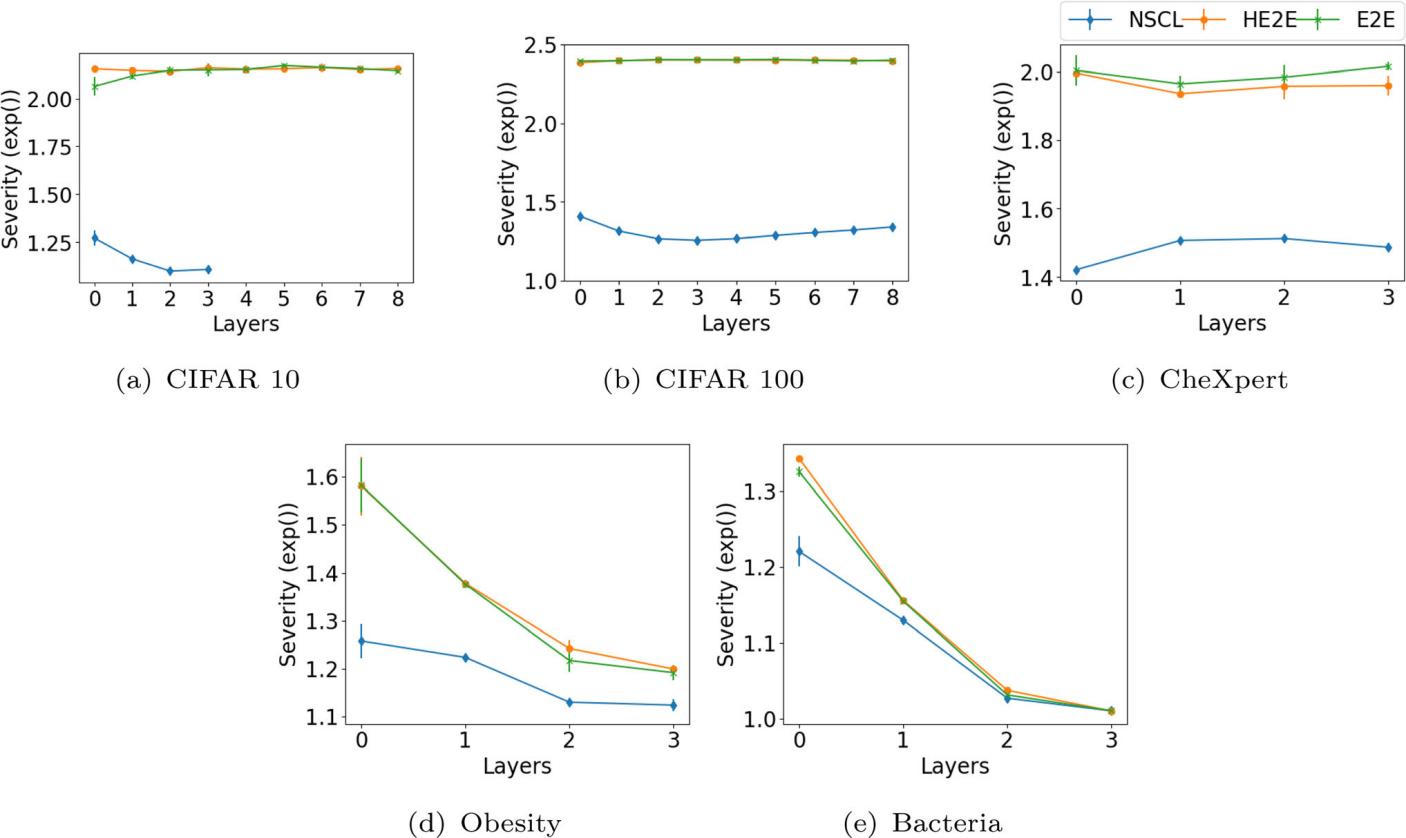
Severity of mistakes made by the different methods. The severity measure penalises confusions between semantically dissimilar classes. Hence, the lower the score, the better the desired performance. While NSCL achieves this objective, E2E and its version with a hierarchical loss does not

### Severity

Figure 4 shows misclassification severity of the three methods on the five problems. As this measure penalises confusion outside semantic groups in class hierarchies, lower values indicate better performance. We see that, evaluated at different layers, NSCL yields lower severity than E2E or HE2E. The hierarchical loss we induce via the regression loss to match image (input) and textual (class labels) information has little effect in E2E training, whereas the nested cascade learner captures such information as desired. It is worth noting that since all three models achieve nearly $100\%$ accuracy on the Bacteria dataset, the errors made by the models are negligible, resulting in minimal severity differences among them (as illustrated in Fig. 4e). In section A.1 (see Figure A3), we give more results comparing different variants of this setting. The overall outcome is that critical errors in a diagnostic setting can be minimised by constraining them to semantically similar groups.

### Saliency maps of image features

**Fig. 5:**
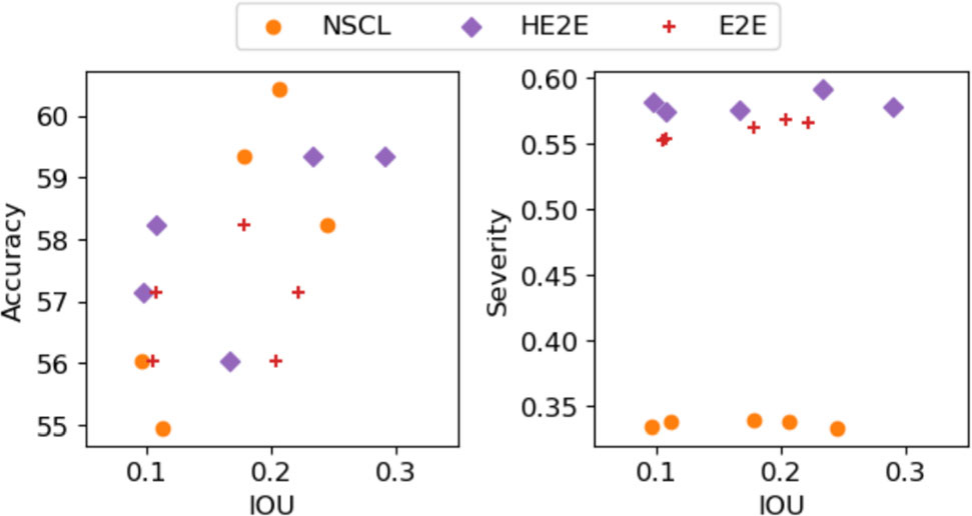
Variation in accuracy, severity and overlap between saliency map and human annotated discriminant locations across five independent runs of the models. We see that in terms of severity, variability in results is very low, making it a more stable measure of performance even though the runs produce their classification decisions based on different localisations

We then compared how peak regions of saliency maps derived from network decisions overlapped with regions of CheXpert images annotated by experts as regions of interest for particular diseases. As often done in the literature, this comparison is done using the IOU as a measure. In Fig. 5, we plot five different runs of the three models and visualise the changes they show in terms of accuracy, severity and IOU, the only difference between runs being random initialisation of network weights. We note that the usual measures of performance (accuracy and IOU) vary substantially across different runs, contributing to the so-called epistemic uncertainty [35] in performance. However, in terms of severity, errors are scored in a more meaningful way, models are far more stable. Comparisons of more models are illustrated in Figures A4 and A6 in section A, where we further compare our learning mechanisms with benchmark results obtained using masks from the large-scale DenseNet121 [15] (approximately 5.3*M* more parameters than our NSCL models) trained for a ten-class binary classification task instead of our six-class classification task. Overall, NSCL exhibits comparable median IOU values over five runs to DenseNet121 across all pathologies; however, DenseNet121 demonstrates higher uncertainty in its IOU estimates from only one run.

### Ablation experiments

To gain a quantitative understanding of the roles of the different components of our approach, we conducted ablation studies: with and without semantic information; tuning the relative weights between regression and classification losses (Eq. 2); and different ways of deriving the class hierarchies.

**Table 1:** Effect of an ablation study, with and without the semantic component. We see a sharp reduction in severity going from NCL and NSCL, showing the importance of semantics in our setting, whereas the reduction in E2E by incorporating a hierarchical loss is minimal

Severity$\downarrow$				
Datasets	Cascade		E2E	
	NSCL (w)	NCL (w/o)	HE2E (w)	E2E (w/o)
CIFAR 10	$\textbf{0}.10_{\pm 8.1e-5}$	$0.76_{\pm 1.8e-5}$	$0.77_{\pm 8.9e-6}$	$0.76_{\pm 4.0e-5}$
CIFAR 100	$\textbf{0}.29_{\pm 1.3e-5}$	$0.88_{\pm 3.2e-6}$	$0.87_{\pm 1.2e-5}$	$0.88_{\pm 4.4e-7}$
CheXpert	$\textbf{0}.40_{\pm 8.3e-5}$	$0.69_{\pm 9.2e-5}$	$0.67_{\pm 2.3e-4}$	$0.70_{\pm 3.9e-5}$

Accuracy				
Datasets	Cascade		E2E	
	NSCL (w)	NCL (w/o)	HE2E (w)	E2E (w/o)
CIFAR 10	$84.44_{\pm 1.88}$	$84.01_{\pm 0.38}$	$85.12_{\pm 0.25}$	$83.09_{\pm 5.38}$
CIFAR 100	$62.22_{\pm 0.09}$	$63.36_{\pm 0.02}$	$60.20_{\pm 0.35}$	$53.86_{\pm 0.19}$
CheXpert	$59.78_{\pm 3.19}$	${60.07_{\pm 1.88}}$	$58.02_{\pm 1.64}$	$56.92_{\pm 0.68}$

**Semantic information** Table 1 compares performances on three problems of NCL with NSCL. The expectation here is that the severity of performance will decrease as a result of injecting semantic information, all other conditions remaining the same. In terms of overall accuracy, however, we would expect little difference. This is indeed the case in the results shown in Table 1. For E2E learning semantic information is injected via the combined loss function. While this does not achieve a better distribution of errors, it does not make performance worse by either of the measures (the differences not being significant). This suggests that we are achieving better success in injecting semantic label information via a cascade learning setting, than with the classic E2E learning.

**Table 2:** Change in performance with tuning of the relative weighting between classification and regression weights (Eq. 2). We note that $\alpha$ and $\beta$ can be tweaked as hyperparameters to achieve an optimal balance

${\alpha }$	${\beta }$	CIFAR 10		CIFAR 100		CheXpert	
		Accuracy$\uparrow$	Severity$\downarrow$	Accuracy$\uparrow$	Severity$\downarrow$	Accuracy$\uparrow$	Severity$\downarrow$
0	1	83.89 ± 0.97	$0.103_{\pm 4.7e-3}$	60.92 ± 0.51	$0.296_{\pm 2.2e-3}$	$56.26_{\pm 1.64}$	$0.399_{\pm 5.0e-4}$
0.2	0.8	82.94 ± 0.59	$0.105_{\pm 4.0e-3}$	61.13 ± 0.04	$0.299_{\pm 3.7e-3}$	$56.92_{\pm 1.64}$	$0.394_{\pm 2.0e-4}$
0.4	0.6	83.03 ± 1.38	$0.110_{\pm 7.0e-3}$	61.41 ± 0.20	$0.296_{\pm 3.6e-3}$	$58.02_{\pm 0.68}$	$0.406_{\pm 1.3e-4}$
0.5	0.5	82.16 ± 1.50	$0.113_{\pm 3.2e-3}$	61.43 ± 0.19	$0.292_{\pm 4.8e-3}$	–	–
0.6	0.4	81.70 ± 0.11	$0.111_{\pm 3.3e-3}$	61.41 ± 0.33	$0.293_{\pm 2.7e-3}$	$57.80_{\pm 2.22}$	$0.412_{\pm 4.6e-5}$
0.8	0.2	81.92 ± 1.90	$0.125_{\pm 4.6e-3}$	**62.65** ± **0.23**	$\mathbf{0.286_{\pm 2.2e-3}}$	$52.09_{\pm 8.02}$	$0.421_{\pm 2.4e-4}$
2	1	–	–	61.79 ± 0.06	$0.294_{\pm 1.8e-3}$	$58.02_{\pm 6.47}$	$0.404_{\pm 2.0e-4}$
3	1	84.19 ± 0.75	$0.107_{\pm 8.1e-3}$	61.79 ± 0.06	$0.296_{\pm 3.6e-3}$	$\mathbf {59.78_{\pm 3.19}}$	$0.397_{\pm 3.5e-5}$
4	1	84.40 ± 1.91	$0.102_{\pm 5.7e-3}$	62.22 ± 0.09	$0.294_{\pm 4.4e-3}$	$59.34_{\pm 1.93}$	${\textbf {0.393}}_{{\pm } {\textbf {2.0e-4}}}$
5	1	84.70 ± 0.08	$0.103_{\pm 1.7e-3}$	–	–	$53.85_{\pm 2.42}$	${0.429_{\pm 1.6e-2}}$
6	1	**85**.**69** ± **0**.**23**	$0.101_{\pm 3.1e-3}$	–	–	$56.04_{\pm 5.64}$	${0.432_{\pm 3.0e-3}}$
7	1	85.47 ± 0.01	$\mathbf {0.098}_{{\pm } {\textbf {1.4e-3}}}$	–	–	$54.21_{\pm 5.10}$	${0.427_{\pm 6.2e-3}}$
8	1	85.25 ± 0.14	$0.101_{\pm 4.9e-3}$	–	–	–	–

**Regression weight** We then systematically changed the relative weights between the regression and classification loss terms to explore if these hyperparameters ought to be optimised, in the NSCL setting. Table 2 shows variation in results of accuracy and severity showing there is scope for such optimisation treating the relative weights as hyper-parameters of the approach. Note also the uncertainty in figures, with accuracy showing far higher variation than severity across trials, a point made earlier in reference to the saliency map results of Fig. 5.

**Fig. 6:**
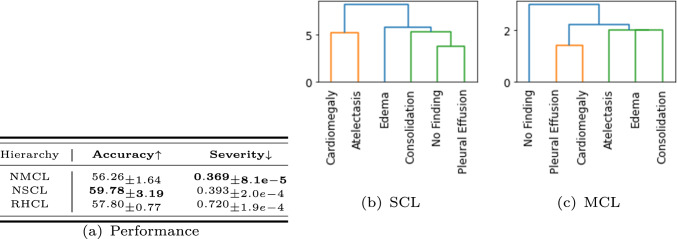
Difference in performance of two ways of establishing a hierarchy of classes. Dendrogram (**b**) is semantic cascade learning obtained by clustering labels, while (**c**) is derived from the directed acyclic graph arising from prior knowledge of the diseases. We also compare a partitioning of the classes into a random dendrogram of the same group sizes and quote the results (RHCL).

**Hierarchy type** And finally, we compared different ways of setting up class hierarchies, results of which are shown in Fig. 6a. Here, we compare an NSCL model in which the hierarchy is derived by clustering of labels (shown in Fig. 6b) against one in which the hierarchy comes from prior medical knowledge (shown in Fig. 6c), made available in the CheXpert dataset in the form of a directed acyclic graph. Interestingly, the dendrogram from clustering differs from domain knowledge, specifically the “No Finding” group does not separate out from any of the diseases as it should. This does point to potential limitations of clustering class labels taken in isolation and using their vector representations obtained via self-supervised learning. Despite this difference, both models exploiting class hierarchies give low severity scores. To calibrate, we also compared a random grouping with the same membership sizes, while achieving comparable accuracy of classification, which does not yield low severity scores (see Figures A2 and A3 in appendix). This points to semantic information being systematically exploited in our learning architecture.

**Hierarchical loss** In addition to the training mechanisms, we also consider studies that proposed Hierarchical Cross-Entropy Loss (HXEloss) for facilitating “better mistakes” as introduced by Srivastava and Mishra [19] and Bertinetto et al. [20]. As presented in Tables A1 and A2, integrating HXEloss into the training of E2E models does not result in significant improvements in prediction accuracy compared to standard E2E training.

On the Obesity dataset, the E2E model with HXEloss demonstrates substantially lower accuracy compared to our proposed NSCL model, under otherwise similar settings. However, E2E with HXEloss exhibits improved stability, as reflected by lower variance in cross-validation results. In terms of severity reduction, the use of HXEloss yields negligible improvements over traditional E2E models, suggesting its limited utility in addressing severe misclassification. This finding is consistent with the observations given by Srivastava and Mishra [19], who noted that while HXEloss aims to prioritise semantically meaningful errors, its impact on overall prediction accuracy and the mitigation of severe misclassification remains limited in certain contexts.

## Discussion

The proposed NSCL method, trained in a stage-wise manner, leverages the semantic hierarchy to structure the model’s learning process. This approach raises important considerations regarding computational complexity, particularly as the number of classes increases. The computational complexity of the NSCL approach is linear (${\mathcal {O}}(L \cdot C)$) with respect to the number of classes *C* and the levels of the hierarchy *L*.

Such a structure ensures that the model remains scalable as datasets expand, making it computationally feasible for large-scale classification tasks. The flexibility in adjusting the hierarchy depth *L* further enhances the efficiency of the model, as the granularity of the hierarchy can be tailored based on the complexity of the task at hand. This offers a trade-off between computational cost and model performance, allowing for optimisations in resource-constrained environments.

However, the determination of an optimal number of hierarchy levels remains an open question. Previous work on hierarchical learning (e.g. [19, 20]) suggests that while deeper hierarchies may allow for more precise classification, they also introduce additional computational costs. A systematic approach to finding the optimal hierarchy depth, possibly guided by task-specific requirements, is needed for further development of NSCL models.

Another area for future exploration is the alignment of features extracted at each stage of the cascade. As the model progresses through stages, the relationship between superclasses and subclasses becomes increasingly complex. Misalignments in feature extraction between these levels can introduce bias, potentially hindering the model’s ability to make accurate predictions. Addressing this issue through methods such as feature re-alignment or domain adaptation [36] could lead to more robust and interpretable models, particularly in complex and imbalanced datasets [19, 24].

Curriculum Learning [37] is indeed a tempting parallel approach to consider, in that the aim is to learn from easy examples first and then on progressively harder ones. Ours is a different philosophy, i.e. to learn from broader (hence easier) tasks first and then progressively finer (hence harder) tasks. Curriculum learning requires an independent way of distinguishing between easy and hard examples, which is not easy to obtain, whereas our approach focuses on semantic information contained in the description of the class labels. While they are different, we would consider future work on exploring use cases in which combining them may offer an advantage.

## Conclusion

In this paper, we address the issue of varying class error severity which is especially important in applications such as medicine where some types of errors may not be important but others can be highly significant. We show how semantic information contained in class labels of multi-class classification problems could be exploited in a coarse-to-fine setting of pattern recognition, grouping semantically similar classes in early stages and finer classification deeper in a layer-wise trained network. We use both image and tabular datasets to illustrate the nested semantic cascade learning methodology. Our use of semantic similarity, which is derived either from prior knowledge or by clustering distributed vector representation of class labels, in effect a proxy for costs of misclassification between classes, should they be available in a practical setting. The metric we define, severity, shows the approach of nested cascade models exploiting such class information far better than vanilla E2E training or E2E boosted by a hierarchical loss. Ablation studies we performed, and the loss function that integrates classification and regression loss, provide substantial additional evidence of this. Deployed systems, be they in medical diagnostics or broader computer vision applications will benefit from this framework, by concentrating on misclassification errors that actually matter.

## Supplementary Information

Below is the link to the electronic supplementary material.

Supplementary file 1 (pdf 3525 KB)

## Data Availability

All data are publicly available. CIFAR 10 and CIFAR 100 datasets can be downloaded from https://www.cs.toronto.edu/~kriz/cifar.html. CheXpert dataset can be downloaded from https://stanfordmlgroup.github.io/competitions/chexpert/. Obesity dataset can be downloaded from https://archive.ics.uci.edu/dataset/544/estimation+of+obesity+levels+based+on+eating+habits+and+physical+condition. Bacteria dataset can be downloaded from https://www.kaggle.com/code/samuelcortinhas/tps-feb-22-model-for-train-test-drift.
