# Supplementary material for: Balancing misclassification errors in image-based inference using problem domain semantics and a nested cascade architecture
Source: Neural Comput Appl. 2025 Sep 13;37(31):26021–36. doi: 10.1007/s00521-025-11613-8 (PMC12535507; doi:10.1007/s00521-025-11613-8)
Supplement: Supplementary file 1 — (pdf 3525 KB) [file 521_2025_11613_MOESM1_ESM.pdf]

Appendix A Supplementary material

Figure A1 provides a visual representation that highlights the distinctions between three mechanisms: End-to-End (E2E), Cascade Learning (CL) and Semantic Cascade Learning (SCL). By examining this figure, we can gain a deeper understanding of how these mechanisms differ in their approach to solving various problems.

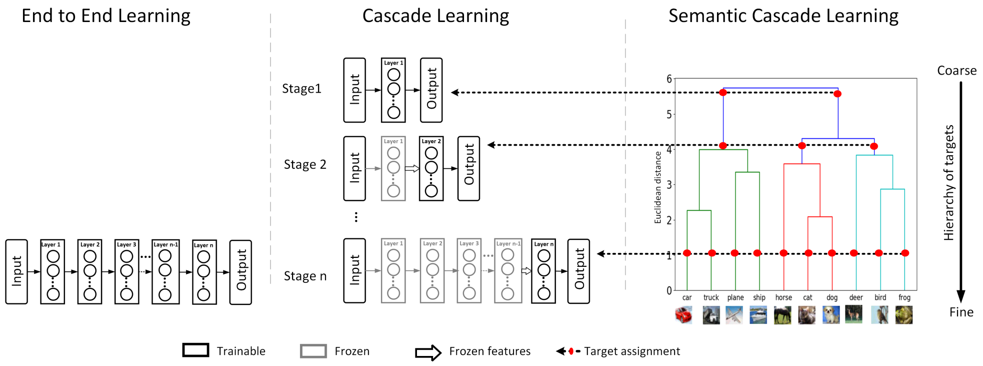

Fig. A1: Illustration of the learning mechanism (similar to Figure 1 in main text). The same network architecture is used for E2E, CL, and SCL. In E2E, all parameters are updated simultaneously at every gradient step. CL updates parameters in stages, freezing the weights learned by previous layers. In nested models, parameters of previous layers are also updated.

A.1 Accuracy and Severity of Variants of the Approach

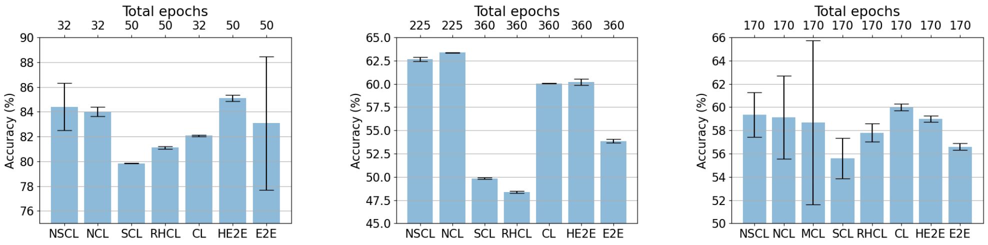

(a) CIFAR 10

(b) CIFAR 100

(c) CheXpert

Fig. A2: Comparative analysis of performance across eight training mechanisms on three datasets. Each bar represents the average classification accuracy and uncertainty of each learning mechanism, calculated over five runs on one dataset. The upper axis indicates the required training epochs for each learning mechanism.

Extending on the three methods compared in the main text (E2E, HE2E and NSCL in section 3.4 and section 3.2.1), here we report results of other variants of the framework. The five additional variants are: Nested Cascade Learning (*i.e.* NSCL without use of semantic information), Semantic Cascade Learning which does not do nesting in its update (SCL), A system that groups classes into a random hierarchy (RHCL) and vanilla Cascade Learning (CL). Additionally, for the medical problem, we report a version that uses a hierarchy derived from prior clinical knowledge (MCL). Accuracy results for these variants are shown in Figure A2 and the corresponding severity results in Figure A3. While there is variation in accuracies across different models, we note that the nested cascade models are competitive (or outperform) the E2E approaches in these problems. More importantly, consistent with the purpose of the present study, the use of semantic information combined with nested cascade training offers the expected advantage in terms of the severity of misclassification.

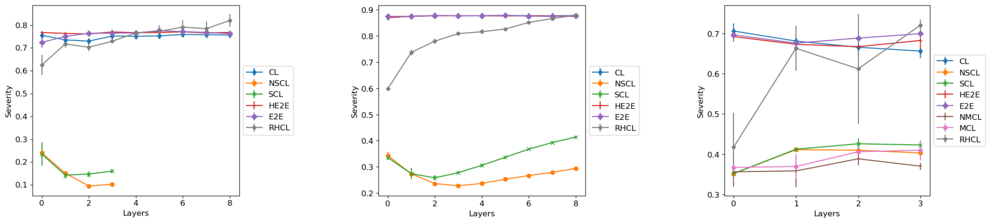

(a) CIFAR 10 (b) CIFAR 100 (c) CheXpert

Fig. A3: The severity of mistakes across multiple training mechanisms on three datasets was compared. For each dataset, each line with an error bar represents the progression of mistake severity from early to later learning stages of a learning mechanism, averaged over five runs. Significant differences were observed between learning mechanisms with and without semantic cascade learning across all three datasets.

A.2 Saliency Maps of Learning Mechanisms

While accuracy and severity serve as metrics for evaluating the learning capability of models, the interpretability of information extracted by these models remains ambiguous. To address this issue, we utilize a saliency map to visualize the regions that contribute to predictions from different learning mechanisms. We sought assistance from a professional radiologist who marked the pathology regions in each image using rectangles. Comparing these maps with annotations provided by the radiologist reveals that most saliency maps exhibit reasonable overlapping regions with annotations located within lung areas. Notably, detecting Cardiomegaly and Consolidation poses a challenge as there is minimal overlap between saliency maps generated by various learning mechanisms and radiologist annotations. However, it is intriguing to observe that when detecting Cardiomegaly, saliency maps primarily emphasize the space within the chest rather than focusing on the heart itself. This results in a lower rate of overlapping for Cardiomegaly which is defined as an increased cardiothoracic ratio

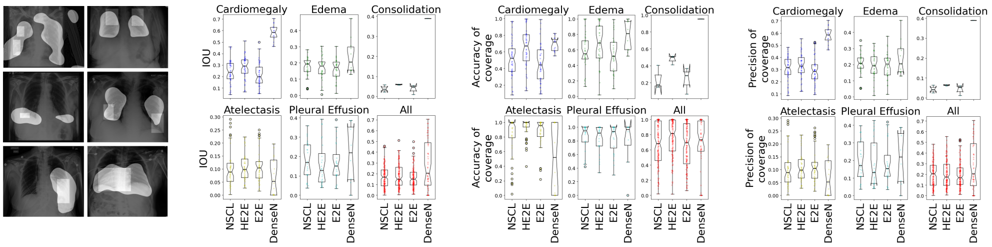

(a) Examples (b) IOU (c) Accuracy of Coverage (d) Precision of Coverage

Fig. A4: Overlaps between regions of interest from saliency maps and clinical annotations in CheXpert data. In addition to the models considered previously, we also include DenseNet121 as used in Saporta et al. [15]. (a) examples of masks obtained by thresholding saliency maps and annotations; (b) variation in IOU across the different methods; (c) rate of overlapping regions defined as accuracy of coverage; and (d) precision of coverage. Note DenseNet121 is an architecture substantially larger in number of parameters (5.3M more parameters) than our architectures.

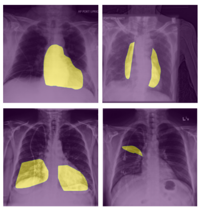

Fig. A5: Examples of annotations from the CheXpert dataset given by Saporta et al. [15].

greater than or equal to 50% on a posterior-anterior projection of chest radiography [38]. This phenomenon suggests that computers and human radiologists may exhibit divergent attentional patterns when recognizing similar diseases; nevertheless, both perspectives hold validity.

The evaluation of saliency maps provided by all learning mechanisms based on our annotations and those of Saporta et al. [15] is further demonstrated in Figure A4 and A6, respectively, as shown in Figure A5. The hit rate, as defined by Saporta et al. [15], assesses the probability of the darkest point within each saliency map falling within the annotated lesion region. DenseNet121 exhibits remarkable performance in highlighting Cardiomegaly and Consolidation under IOU evaluation, but shows relatively lower accuracy in localizing Edema and Atelectasis. Moreover, DenseNet121 demonstrates a lower hit rate for identifying lesion regions associated with Edema, Atelectasis, and Pleural Effusion; however, it achieves a higher hit rate for Cardiomegaly and Consolidation.

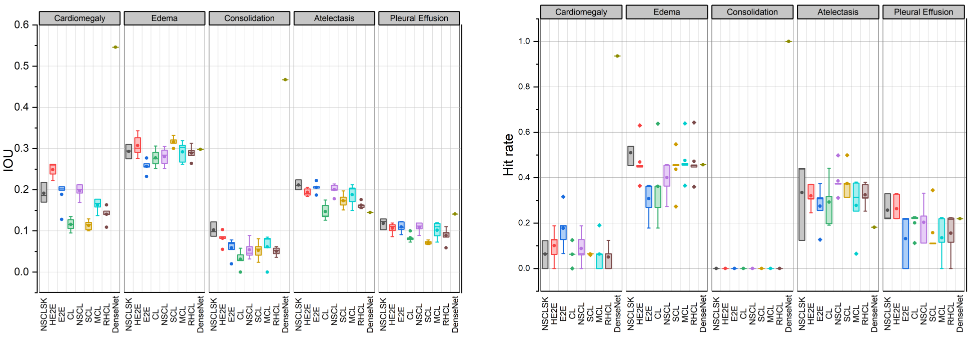

(a) IOU (b) Hit Rate

Fig. A6: Comparisons of IOU and hit rate among learning mechanisms based on annotations given by Saporta et al. [15]. The performance of our simple models, in terms of IOU and hit rate evaluation metrics, is comparable to that of the large-scale DenseNet121 model, except for Cardiomegaly and Consolidation.

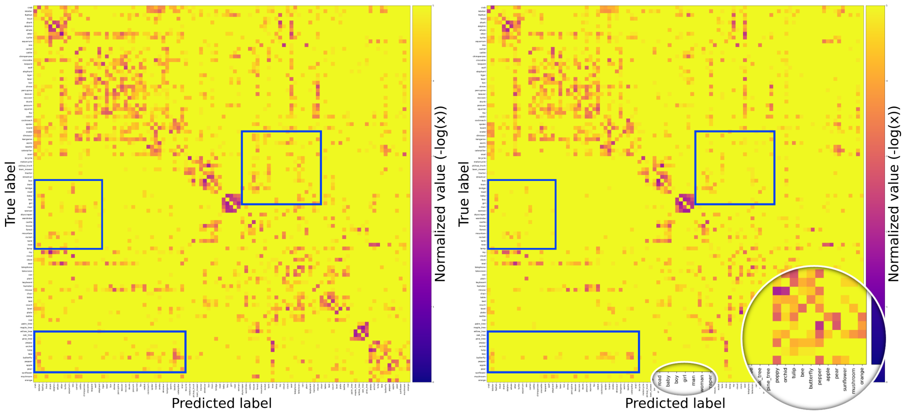

(a) E2E (b) NSCL

Fig. A7: Comparing confusion matrices of E2E and NSCL models on the CIFAR100. A zoom-out version of Figure 3 in the main text.

A.2.1 Visualization

To better understand the distribution of correctly classified data points and their relationship to the semantic hierarchical structure, we employed Uniform Manifold Approximation and Projection for Dimension Reduction (UMAP) [39]. This technique was used to project the latent representations of inputs from the layer preceding the prediction layer, enabling us to visualize the distribution of correctly classified points

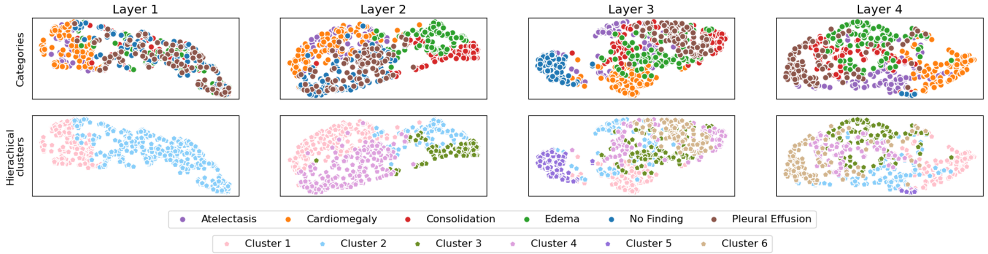

(a) NSCL

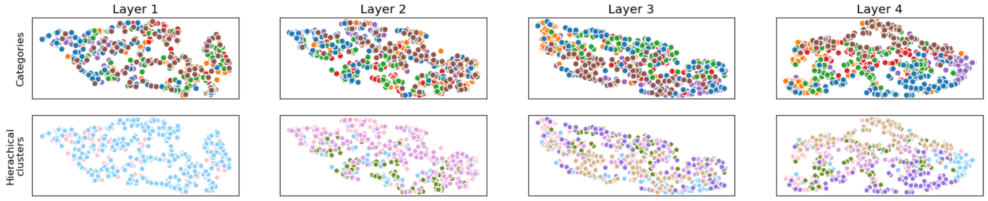

(b) E2E

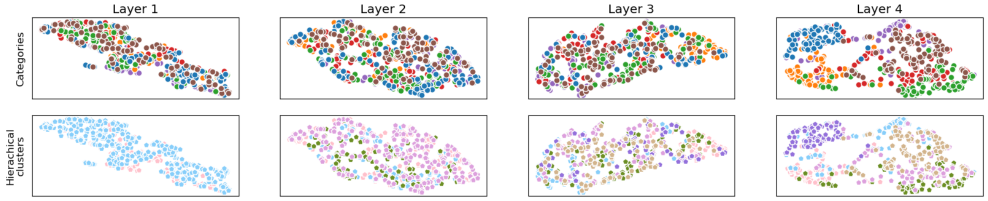

(c) HE2E

Fig. A8: Illustration of the distribution of correctly classified points across different models. In each subplot, the first row depicts points colored by their original categories, while the second row shows the same points colored according to clusters defined by the semantic hierarchy in the CheXpert dataset.

given by each model. Figure A8 shows the distribution of projected representations from three learning mechanisms. The latent representations from the NSCL model exhibit clear boundaries between coarse classes at higher hierarchical levels (as shown in Figure A8(a)). In contrast, the corresponding representations from the E2E and HE2E models show significant overlap, with points from different border clusters mixing together (as shown in Figure A8(b) and A8(c)).

A.3 Details of Tabular Datasets

As mentioned in the main text, this work also explores the tabular datasets Obesity and Bacteria. Figure A9 illustrates the hierarchical label structure for both datasets. The semantic embeddings of the labels are generated using a pre-trained word-to-vector model (Wiki News 300d 1M). These embeddings are then clustered based on Ward's

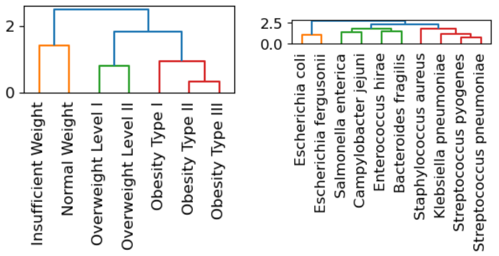

(a) Obesity (b) Bacteria

Fig. A9: Illustration of classes hierarch generated based on semantic embeddings of labels.

distance to obtain the hierarchical structure. The number of classes of a four-layer model on the Obesity dataset is two, three, four, seven, and on the Bacteria dataset is two, four, eight and ten. The node of each superclass can be selected by the program automatically based on the corresponding dendrogram plot.

A.3.1 Comparison of evaluation metrics on tabular datasets

Tables A1 and A2 present a comparison of evaluation metrics, including accuracy, precision, recall, F1 score, and severity, for the two datasets. NSCL demonstrates superior performance compared to the E2E model across all metrics for both datasets. While the accuracy of classification is close to 100%, NSCL and HE2E show similar severity.

Table A1: Comparison of evaluation metrics on Obesity dataset.

**Model**	**Accuracy ↑**	**Precision ↑**	**Recall ↑**	**F1 score ↑**	**Severity (exp()) ↓**
NSCL	**83.81**$_{\pm \mathbf{0.76}}$	**0.89**$_{\pm \mathbf{9.0e-4}}$	**0.86**$_{\pm \mathbf{4.2e-4}}$	0.86$_{\pm 6.1-4}$	**1.12**$_{\pm \mathbf{0.013}}$
HE2E	74.17$_{\pm 1.16}$	0.85$_{\pm 4.4e-3}$	0.79$_{\pm 7.9e-5}$	**0.87**$_{\pm \mathbf{1.9e-4}}$	1.24$_{\pm 0.008}$
E2E	75.83$_{\pm 1.40}$	0.87$_{\pm 4.2e-4}$	0.79$_{\pm 2.3e-4}$	0.78$_{\pm 3.6e-4}$	1.19$_{\pm 0.016}$
E2E (HXEloss)	73.62$_{\pm 0.39}$	0.81$_{\pm 3.0e-3}$	0.78$_{\pm 2.6e-5}$	0.76$_{\pm 1.7e-4}$	1.17$_{\pm 0.009}$

Table A2: Comparison of evaluation metrics on Bacteria dataset.

**Model**	**Accuracy ↑**	**Precision ↑**	**Recall ↑**	**F1 score ↑**	**Severity (exp()) ↓**
NSCL	98.743$_{\pm 2.9e-4}$	0.988$_{\pm 3.58e-8}$	0.987$_{\pm 3.78e-8}$	0.987$_{\pm 3.84e-8}$	**1.010**$_{\pm \mathbf{2.2e-4}}$
HE2E	98.770$_{\pm 1.9e-3}$	**0.989**$_{\pm \mathbf{1.32e-7}}$	**0.988**$_{\pm \mathbf{1.76e-7}}$	**0.988**$_{\pm \mathbf{1.74e-7}}$	1.010$_{\pm 3.8e-4}$
E2E	98.727$_{\pm 2.2e-5}$	0.988$_{\pm 8.1e-9}$	0.987$_{\pm 9.7e-10}$	0.987$_{\pm 1.56e-9}$	1.011$_{\pm 1.0e-4}$
E2E (HXEloss)	**98.78**$_{\pm \mathbf{2.4e-3}}$	0.87$_{\pm 1.87e-7}$	0.79$_{\pm 2.55e-7}$	0.78$_{\pm 2.51e-7}$	1.010$_{\pm 4.6e-4}$
